# A Residual-Dense-Based Convolutional Neural Network Architecture for Recognition of Cardiac Health Based on ECG Signals

**DOI:** 10.3390/s23167204

**Published:** 2023-08-16

**Authors:** Alaa E. S. Ahmed, Qaisar Abbas, Yassine Daadaa, Imran Qureshi, Ganeshkumar Perumal, Mostafa E. A. Ibrahim

**Affiliations:** 1 College of Computer and Information Sciences, Imam Mohammad Ibn Saud Islamic University (IMSIU), Riyadh 11432, Saudi Arabia; asmohamed@imamu.edu.sa (A.E.S.A.); ymdaadaa@imamu.edu.sa (Y.D.); iqureshi@imamu.edu.sa (I.Q.); gpperumal@imamu.edu.sa (G.P.); meibrahim@imamu.edu.sa (M.E.A.I.); 2 Electrical Engineering Department, Faculty of Engineering at Shoubra, Benha University, Cairo 11629, Egypt; 3 Department of Electrical Engineering, Benha Faculty of Engineering, Benha University, Benha 13518, Qalubia, Egypt

**Keywords:** cardiac health, heart disease detection, convolutional neural, deep learning, electrocardiogram (ECG), feature extraction, residual blocks, long-term short-term memory

## Abstract

Cardiovascular disorders are often diagnosed using an electrocardiogram (ECG). It is a painless method that mimics the cyclical contraction and relaxation of the heart’s muscles. By monitoring the heart’s electrical activity, an ECG can be used to identify irregular heartbeats, heart attacks, cardiac illnesses, or enlarged hearts. Numerous studies and analyses of ECG signals to identify cardiac problems have been conducted during the past few years. Although ECG heartbeat classification methods have been presented in the literature, especially for unbalanced datasets, they have not proven to be successful in recognizing some heartbeat categories with high performance. This study uses a convolutional neural network (CNN) model to combine the benefits of dense and residual blocks. The objective is to leverage the benefits of residual and dense connections to enhance information flow, gradient propagation, and feature reuse, ultimately improving the model’s performance. This proposed model consists of a series of residual-dense blocks interleaved with optional pooling layers for downsampling. A linear support vector machine (LSVM) classified heartbeats into five classes. This makes it easier to learn and represent features from ECG signals. We first denoised the gathered ECG data to correct issues such as baseline drift, power line interference, and motion noise. The impacts of the class imbalance are then offset by resampling techniques that denoise ECG signals. An RD-CNN algorithm is then used to categorize the ECG data for the various cardiac illnesses using the retrieved characteristics. On two benchmarked datasets, we conducted extensive simulations and assessed several performance measures. On average, we have achieved an accuracy of 98.5%, a sensitivity of 97.6%, a specificity of 96.8%, and an area under the receiver operating curve (AUC) of 0.99. The effectiveness of our suggested method for detecting heart disease from ECG data was compared with several recently presented algorithms. The results demonstrate that our method is lightweight and practical, qualifying it for continuous monitoring applications in clinical settings for automated ECG interpretation to support cardiologists.

## 1. Introduction

Millions of people die each year from cardiovascular disorders, which are among the most serious illnesses. A new study estimates that globally, 17.9 million individuals passed away from cardiovascular illnesses in 2019 [1]. This demonstrates how an inadequate detection strategy contributes to the severity of heart disease. Heart attacks and strokes account for the majority of these fatalities [2]. Therefore, in order to provide proper medical care, we need new frameworks for the early identification and diagnosis of heart abnormalities. There are now a number of devices that can monitor human health using wireless sensors, thanks to recent developments in wearable electronics and data-transmission infrastructure [3]. Blood circulation depends on the human heart, which is also where most cardiovascular problems start.

**Figure 1:**
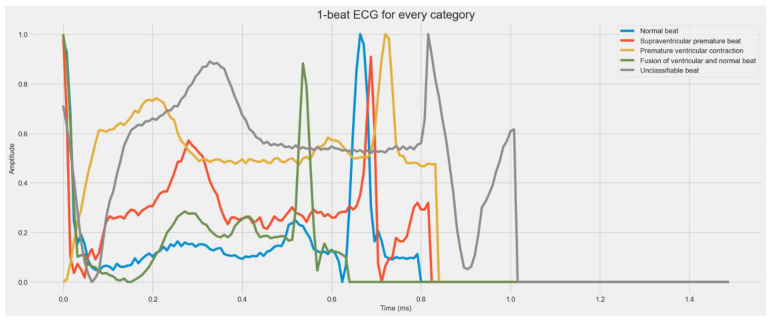
A visual diagram of five stages of heartbeat sounds such as Normal heartbeat, Supraventricular premature beat, Premature ventricular contraction, Fusion of ventricular, and Normal beat, and Unclassifiable beat.

Electrocardiograms (ECGs) can be used to track the heart’s rhythmic activity [4]. The condition of the heart may be quickly diagnosed with an ECG, a non-invasive test. It is often used to check on the heart. It is a tool that captures the electrical impulses the heart generates as it circulates blood throughout the body [5]. Due to the simplicity of testing it provides, the ECG is a widely used medical instrument for monitoring heart rates. The ECG signals must be analyzed with great skill, though. Additionally, interpreting the ECG is particularly difficult since it frequently requires analyzing each heartbeat. The analysis might also contain human mistakes, which is another option. Consequently, it is essential to use an automated computational approach. Arrhythmia is one of the most severe cardiovascular illnesses. The term “arrhythmia” describes deviations from the heart’s normal rhythm or beat rate. Arrhythmia is the medical term for a heartbeat that is abnormally rapid, slow, or irregular [6]. Depending on the patterns shown during the ECG measurements, there are several kinds of arrhythmias. The two significant categories of arrhythmia are morphological and rhythmic. While rhythmic arrhythmia is the condition in which irregular heartbeats have a regular pattern, morphological arrhythmia is defined by the rare occurrence of an irregular heartbeat. Arrhythmias can also be categorized according to where they occur in the heart. As shown in Figure 1, there are five important types of arrhythmia: Normal Beat, Supraventricular Premature Beat, Premature Ventricular Contraction, Fusion of the ventricular and Normal Beat, and Unclassifiable Beat.

In the proposed model, we combine the strengths of residual blocks and dense blocks within a convolutional neural network (CNN). The objective is to leverage the benefits of residual and dense connections to enhance information flow, gradient propagation, and feature reuse, ultimately improving the model’s performance. When residual and thick blocks are used together, the best parts of both are taken advantage of. This improves information flow, gradient stability, and feature reuse, which can lead to better learning of representations and better model performance. The proposed model can be trained using standard optimization techniques, such as stochastic gradient descent (SGD) or the Adam optimizer, along with appropriate loss functions for the specific task (e.g., cross-entropy loss for classification). Regularization techniques like dropout or batch normalization can be applied to prevent overfitting. The proposed Residual-Dense Convolutional Neural Network methodology combines the advantages of residual blocks and dense blocks to enhance the performance of CNN models. This methodology promotes information flow, gradient stability, and feature reuse by integrating residual and dense connections, improving representation learning and model accuracy. This proposed model consists of a series of residual-dense blocks interleaved with optional pooling layers for downsampling. Each residual-dense block incorporates residual and dense connections to facilitate effective feature learning and representation.

The ECG signals are first preprocessed and denoised. It aids in the removal of unimportant noise and mistakes, including contact loss, motion noise, and Gaussian noise. We separate the signals after denoising them to draw attention to the critical call and amplify it. After that, we add new data to the collection to balance the dataset for training and testing and offset the impacts of class imbalance. The expanded dataset is fed through a deep convolutional neural network architecture to extract critical characteristics from ECG signals. These extracted characteristics capture the distinctive elements of the ECG signals that are important for identifying patient arrhythmias. The retrieved features are fed through an RD-CNN classifier to categorize the ECG signals into various arrhythmias. The performance of our model is further optimized via hyperparameter adjustment. To determine the impact of the suggested approach’s different elements on our model’s functionality, we conducted ablation research. We ran extensive simulations on several benchmarked arrhythmia-based ECG signal datasets and assessed many standard performance criteria. The resulting results were evaluated against several modern methods. The collected findings show how well our suggested approach for detecting arrhythmias in ECG data works.

### 1.1. Main Contribution

The major contributions of our work are as follows:

1. We present a deep-learning (DL)-based framework, named RD-CNN, for detecting and classifying five classes of arrhythmia from ECG signals.
2. The combination of residual and dense blocks leverages the benefits of both approaches, promoting effective information flow, gradient stability, and feature reuse, which can lead to improved representation learning and better model performance.
3. The proposed model can be trained using standard optimization techniques, such as stochastic gradient descent (SGD) or Adam optimizer, along with appropriate loss functions for the specific task (e.g., cross-entropy loss for classification). Regularization techniques like dropout or batch normalization can be applied to prevent overfitting.
4. We provide a deep CNN-based feature extraction method and use the deep learning algorithm to categorize the ECG data into the various types of heartbeat. The deep CNN-based strategy combined with the image augmentation technique helps to enhance the dataset’s quality and extract features as efficiently as possible.
5. An ablation study is performed to analyze the effects of the various components of our signal processing techniques on the obtained results. To examine how the various elements of our signal processing approaches affect the outcomes, an ablation study is conducted.

### 1.2. Paper Organization

The remainder of the essay is structured as follows. In Section 2, we discuss the research that has been done in the area of heartbeat identification using ECG data. Section 3 provides a thorough explanation of our suggested technique, a brief explanation of the datasets, and the assessment measures employed. We conducted an experimental investigation, which is demonstrated in Section 4. The research discussions are described in Section 5. Conclusions and suggestions for further development are provided in Section 6.

## 2. Literature Review

In this section, we discuss the research that many researchers have done to improve the identification of ECG-based arrhythmias. The ECG signals have built-in noise, which makes analysis highly challenging. Reading the ECG signals also demands a great deal of skill. Researchers have been inspired by this throughout the years to suggest more precise and automated methods to increase the efficacy and efficiency of ECG signal processing [7]. The majority of current research on improving arrhythmia diagnosis using electrocardiograms falls under the categories of parametric feature-based and signal processing-based research. The next section discusses the work done in various sub-domains.

In [8], the authors introduced a supervised classification method that performed the classification using a support vector neural network (SVNN). The ECG signals were subjected to feature extraction using a wavelet and Gabor filter. They trained SVNN using a genetic approach called Bat Optimization. They attained a classification accuracy of 96.96% using their method. In the research [9], the authors utilized the Support Vector Machine (SVM) to classify the data using the nonparametric power spectral density (PSD) approach to extract features. They chose the SVM’s parameters using Particle Swarm Optimization (PSO). Their method has a 96.06% classification accuracy rate. Using a deep neural network, the authors of [10] divided the ECG data into 13, 15, and 17 groups. For the 13, 15, and 17 classes, they attained classification accuracy of 95.2%, 92.5%, and 91.33%, respectively. They measured a calculation time of 0.015 s on average. With more lessons, their work showed that their performance was declining. Additionally, in [11], the segmented features were retrieved using Dynamic Time Warping (DTW) and Principal Component Analysis (PCA). For the final classification, a Radial Base Function (RBF)-based SVM was fed the collected segmented features. They scored 97.80% accuracy.

On the MIT-BIT arrhythmia database, the authors of [12] suggested a deep learning-based method for identifying arrhythmia from ECG signals and achieved a classification accuracy of 94.2%. A deep convolutional neural network was employed for feature extraction, while a straightforward neural network with backpropagation was used for classification. In [13], the authors suggested a framework for machine learning to extract vital patterns from mobile ECG signals. A three-stage hybrid machine learning framework is proposed for estimating ECG duration associated with cardiovascular disease. Initially, a support vector machine (SVM) is used to recognize the ECG’s unprocessed heartbeats. The authors of [14] also conducted research to classify the arrhythmia signal into one of the 10 disorders, which includes sinus arrhythmia. They classified data using the radial basis probabilistic process neural network (RBPPNN), and their classification accuracy was 75.52%, with the greatest disease-specific accuracy coming in at 86.75%.

The authors used the MIT arrhythmia and MIT supraventricular arrhythmia databases, similar to [15]. The ECG data were divided into five arrhythmia classes using a two-layered, fully linked neural network architecture that was suggested. The architecture’s two levels each had a separate neural network that was completely interconnected. They ran the simulations using the AAMI guidelines and achieved 93.4% accuracy. The authors of [16] used a convolutional neural network architecture to study rhythmic cardiovascular motions in order to identify arrhythmia. They employed Fourier transformations and the dynamic wavelet transform for resampling and denoising throughout the filtering process. They received an F-Score of 82%. They also employed a multilayer perceptron (MLP) [17] to identify arrhythmias from ECG data. They extracted the features using a field-programmable gate array and then reduced the features using DWT. The classification accuracy of their suggested method was 98.3%. A deep deterministic learning (DDL) technique was suggested in the study [18] to detect the various cardiovascular illnesses based on the ECG waves. They employed a synthetic neural network architecture to carry out pattern identification and categorization. The simulations were run on several publicly accessible datasets and ECG samples that the researchers had individually gathered. Their suggested method had a 98% classification accuracy rate overall.

The authors of [19] conducted research to improve the detection of atrial fibrillation in ECG data. Arrhythmias most frequently occur in the form of atrial fibrillation (AF). They suggested using tightly linked neural networks in one dimension for deep learning. According to the experimental findings, the suggested method has a classification accuracy of 99.35%. The authors in [20] described a method to record the rhythmic variations in heartbeat that are symptomatic of different disorders, such as atrial fibrillation. For the purpose of extracting pertinent information from the ECG signals, they used improved QRS complex recognition. They performed feature extraction using nonlinear principal component analysis and employed a radial basis function network (RBFN) for classification, combining neural networks with PCA. According to the authors of [21], an evolving neural system based on the SVM framework can be used to identify irregular heartbeats. The accuracy of their classification of the ECG signals into 17 different cardiac illnesses was 98.85%. For the most part, the authors developed machine learning for the categorization of ECG signals using a dataset from the MIT-BIH arrhythmia database [22].

Recently, the authors proposed [23] a few-shot learning paradigm based on Siamese Convolutional Neural Networks (SCNN) to classify 12-Lead ECG heartbeats using a few training samples with supervised information. Based on a public dataset and the hold-out validation approach, which was used for various combinations of similarity and loss functions, the suggested SCNN model demonstrated accuracy of up to 95%, whereas in [24], ECG signals’ temporal characteristics were first removed, then combined with the original input to provide a time representation input. Then, for feature extraction, a deep-learning network incorporating a convolutional neural network and long short-term Memory was used. Focusing on feature differences required the employment of a simultaneous attention technique. An accuracy of 98.95% was reported by the proposed method in the classification of five classes of heartbeats.

In fact, in [25], the authors summarized the benefits of using all three networks together. For example, autoencoders provide high-level features without the need for preprocessing; random neural networks provide strong generalization and quick training; and RBF neural networks allow you to use what you already know. RR interval-based features and coded features, which are acquired via the autoencoder, are employed instead. We do tests on the MIT-BIH arrhythmia dataset to assess the performance of the suggested system, and we consider the association’s suggestions for the development of medical equipment, which specify five classes of interest. In [26], the authors suggested a model that adapts the classification process and incorporates the trait of patient-specific analysis.

In [27], the modified convolutional network with channel attention (MCC-Net) mechanism was presented. Using the MIT-BIH arrhythmia database, the suggested method obtained 99.98% accuracy (ACC) for five categories. In [28], in comparison to plain-vanilla CNN- and SincNet-based models, the proposed WavelNet-based models demonstrated excellent performance on classifying non-ectopic, supraventricular-ectopic, and ventricular-ectopic beats. In contrast, the authors in [29] were trying to solve the drawbacks of current wearable devices for ECG detection. In this paper, the authors showed how traditional convolutional neural networks, deep neural networks, and hardware acceleration can be used to make a cardiac rhythm abnormality classification model that is light and accurate enough to compete. The MIT-BIH arrhythmia database dataset was used to analyze the architecture, and the results indicated a classification accuracy of 97.69% and a classification time of 0.3 ms for a single heartbeat. In [30], convolutional neural network (CNN) classifiers might offer improved overall accuracy. In this paper, we suggest a CNN-based approach for classifying ECG heartbeats. Based on the MIT-BIH arrhythmia database, our suggested technique had an overall accuracy of 99.43%.

In [31], the authors proposed an optimization phase for the deep CNN model using a brand-new focus loss function to get around this problem. Our suggested strategy obtained 98.41% accuracy, 98.38% F1-score, 98.37% precision, and 98.41% recall. Additionally, our method outperformed current cutting-edge techniques in terms of performance. Moreover, in [32], a unique deep CNN model for reliable heartbeat classification is based on cutting-edge deep learning methods. They reported an accuracy of 99.48%, whereas in [33], a brand-new automated categorization method is suggested. A deep structure with numerous input layers is presented, which is based on long-short-term memory (LSTM) and convolutional neural network (CNN) networks. Based on various heartbeat locations and RR interval properties, four input layers are created. Different strides are used to convolve the first three inputs. Then, the three CNN outputs are combined and sent via an LSTM network. The output is joined with the fourth input after two fully linked layers. The last fully connected layer eventually produces the anticipated label. The MIT-BIH arrhythmia database’s two division algorithms were used to assess the proposed approach. As a result, the technology may be applied in a clinical setting. In [34], in order to automatically categorize the heartbeat of an arrhythmia, a better CNN is presented in this study. All heartbeats are first separated from the original signals. The ECG heartbeats can be fed into the first convolutional layers after segmentation. Each convolution layer in the suggested layout uses kernels of various sizes, fully using the characteristics at various scales. A max-pooling layer came next. The last pooling layer’s outputs are combined and used as the input for fully linked layers. The experiment shows that 99.06% of the heartbeat detection accuracy was achieved. Table 1 summarizes the recent state-of-the-art ECG heartbeat recognition systems.

## 3. Proposed Work

**Figure 2:**
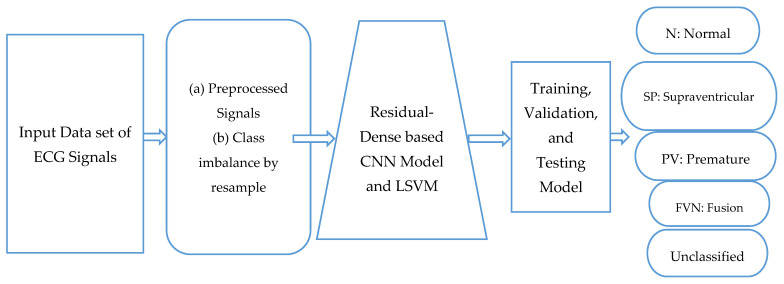
A systematic flow diagram of proposed RD-CNN architecture to classify five classes of cardiac disease heartbeats.

This part presents an example of our suggested deep-learning approach for identifying arrhythmias from ECG information. The five steps of the proposed algorithm are as follows: the ECG signals are denoised, data are augmented, features are extracted using a residual-dense convolutional neural network, and LSVM is used for classification. We denoise the ECG data to produce cleaner waves without extraneous noise. Further enhancements are made to the preprocessed photos to correct the sample imbalance. The deep Convolutional Neural Network (CNN) architecture is then given the enhanced dataset to extract features from. The LSVM algorithm receives the retrieved features and performs the final classification. We suggested deep learning (Residual-CNN) and LSVM-based frameworks for arrhythmia identification of ECG data are shown in Figure 2 as a flow diagram. The following is a description of each phase.

### 3.1. Acquisitions of Dataset

We utilized two datasets, which are publicly available, to perform simulations of the work. It also exemplifies the many assessment measures we employed to assess the effectiveness of the work we had suggested. The following is a description of the dataset. These two benchmarked datasets have been heavily referenced in recent literature in order to carry out the simulations. The MIT-BIH Arrhythmia Dataset [22,23] and the PTB Diagnostic ECG Dataset [23,24] are the datasets that we utilized. Below is a quick explanation of the datasets:

Table 2 shows the main heartbeat types included in the MIT-BIH Arrhythmia and PTB Diagnostic ECG Datasets related to five classes of heartbeats. The MIT-BIH Arrhythmia dataset contains 48 half-hour segments of two-channel ambulatory ECG recordings as data samples. The information is accessible to everyone at physionet.org. It includes the following five classifications: Normal Beat, Supraventricular Premature Beat, Premature Ventricular Contraction, Fusion of the ventricular and Normal Beat, and Unclassifiable Beat; whereas in PTB’s Diagnostic ECG Dataset’s sample signals, the information is accessible to everyone at physionet.org. The main categories of heartbeats are listed in Table 2, and the sample signals for each category are displayed in Figure 1 of the PTB and MIT-BIH Diagnostic ECG Datasets for each class.

### 3.2. Denoising Pre-Process for ECG Signals

The denoising process is integrated as a preprocessing step to improve the quality of the input data. Denoising ECG signals is a crucial step in improving the accuracy of arrhythmia detection, as it helps remove unwanted noise and artifacts that can hinder the interpretation of the underlying cardiac activity. In the context of the Residual-Dense Convolutional Neural Network (RD-CNN) model for arrhythmia detection, a denoising process is likely implemented to enhance the quality of ECG signals before they are fed into the model. Let us delve into a general denoising process and provide an example of how it could handle various types of noise and errors commonly encountered in ECG signals, including contact loss, motion noise, and Gaussian noise.

Let us consider a scenario where an ECG recording is affected by contact loss, motion noise due to patient movement, and Gaussian noise. The following steps demonstrate how the denoising process might be applied:

(a) Contact Loss Handling: Identify segments of the ECG signal where contact loss occurs. Interpolate missing data points within these segments using techniques like linear or cubic interpolation.
(b) Motion Noise Reduction: Apply adaptive filtering to suppress motion artifacts. This involves estimating the motion-induced noise profile and then subtracting it from the original signal.
(c) Gaussian Noise Removal: Utilize a wavelet denoising approach. Apply a discrete wavelet transform to the signal, threshold the wavelet coefficients to remove noise components, and then reconstruct the denoised signal.

By combining these techniques, the denoised ECG signal becomes more suitable for accurate arrhythmia detection by the RD-CNN model. The denoising process ensures that noise and errors introduced by contact loss, motion, and Gaussian interference are effectively addressed, leading to improved performance in arrhythmia detection.

### 3.3. Class Imbalance by Resample

**Figure 3:**
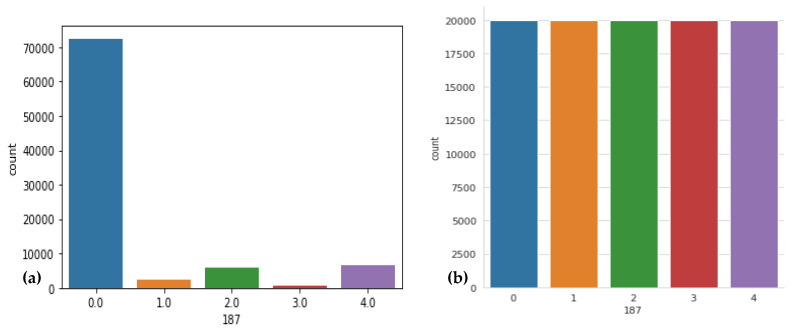
Sample distribution before and after resizing was performed, where (**a**) shows the original dataset with respect to heartbeat class, (**b**) displays the data distribution after resizing performed, where 0 label shows the normal beat, 1 shows supraventricular premature beat, 2 label shows premature ventricular contraction, 3 label shows fusion of ventricular and normal beat, and 4 label shows unclassifiable beat.

Downsampled BIDMC to 128 Hz to fit MIT-BIH, filter for only (N) normal beats, each heartbeat being z-normalized; and since each subject has a large amount of up to 70,000 beats, the study extracted a beat every 5 s. The distribution of classes in Figure 3a demonstrates the imbalance in the MIT-BIH and PTB datasets. As a result, the class with the most samples is biased, and the classification performance suffers to categorize five classes of downsampling while the number of samples in the other classes is increased by upsampling. Table 3 displays the class distributions prior to and following these operations. From Table 3, it is clear that there is a huge class imbalance in this dataset. The model can easily diminish the loss by ignoring everything other than class 0. Next, we take the overrepresented class (N) and sample 5000 samples randomly. These will be used for training, and the rest will be ignored. The number of ECG signals falling within each type varies significantly. A visual result of this technique is displayed in Figure 3b.

In the context of addressing class imbalance, an important observation is that the model might tend to prioritize or focus on the majority class (Class 0), leading to potential performance issues and biased results. To counter this, a strategic approach is taken to balance the dataset and improve the model’s overall performance.

The first step involves focusing on the overrepresented class, which is referred to as Class 0. The aim is to prevent the model from disproportionately favoring this class and ignoring the other classes. By doing so, the model’s loss function encourages all classes to be considered equally, thus promoting more balanced learning. Moving forward, attention is directed towards handling the overrepresented class. From this class, a subset of 5000 samples is randomly sampled. These samples are meticulously chosen to maintain a representative and unbiased distribution. This subset serves as the training data, enabling the model to learn from a more diverse range of examples within the overrepresented class.

Concurrently, the remaining data points from the overrepresented class are deliberately excluded from the training process. This selective sampling strategy is designed to strike a balance between preventing class bias and ensuring efficient and effective model training. This approach demonstrates a thoughtful method to manage class imbalance and enhance model training. By diminishing the influence of the majority class and strategically sampling from the overrepresented class, the resulting dataset provides the model with a more equitable representation of different classes. This measured approach not only fosters improved classification performance but also maintains fairness and accuracy in the model’s predictions across all classes. In order to reduce overfitting and bias, the class with the most samples is downsampled, while the number of samples in the other classes is increased by upsampling.

### 3.4. Feature Extraction Using Residual-Deep CNN Model

Instead of manually collecting the necessary characteristics from the ECG signal dataset that was acquired in the previous phases, the salient features are extracted from the images using a Residual-Deep CNN model architecture. Convolutional layers, each followed by a max pooling layer, make up the CNN architecture used in this work. We use a convolutional neural network (CNN) model to combine the benefits of dense and residual blocks. The benefits of residual and dense connections are integrated to enhance information flow, gradient propagation, and feature reuse, ultimately improving the model’s performance. This proposed model consists of a series of residual-dense blocks interleaved with optional pooling layers for downsampling. Each “residual-dense” block has both “residual” and “dense” connections. This makes it easier to learn and represent features from ECG signals, and each heartbeat is put into one of five classes.

**Figure 4:**
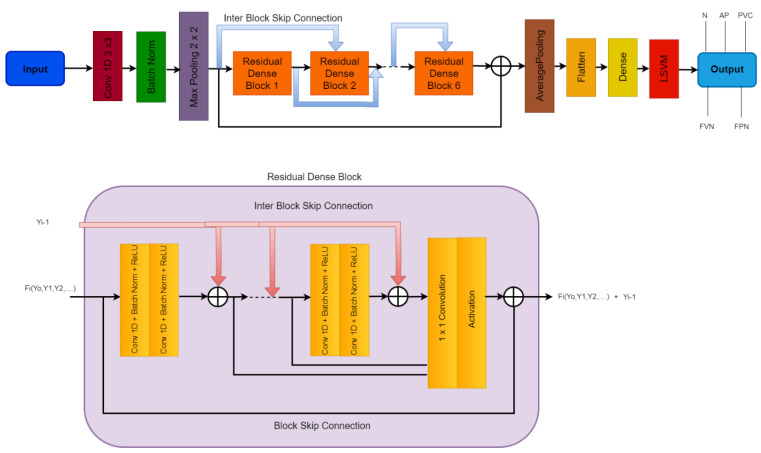
A systematic flow diagram of the proposed RD-CNN architecture to classify five classes of heart diseases, where N: Normal Beat, AP: Supraventricular Premature Beat, PVC: Premature Ventricular Contraction, FVN: Fusion of Ventricular and Normal Beat, and FPN: Unclassifiable Beat are classes of cardiac disease heartbeats.

The Residual-Deep (RD-CNN) CNN architecture utilized to extract features from the ECG data is shown in Figure 4. The numerous hyperparameters of the RD-CNN architecture shown in Figure 4. All of the key elements from the ECG signals are extracted using the deep CNN architecture. Additionally, while retaining a manageable computational cost, an appropriately deep CNN architecture guarantees the dependability of the extracted features. In the following stage, the RD-CNN algorithm makes use of these retrieved characteristics.

In order to create the training and testing datasets, we first divided the complete dataset into an 80:20 ratio, retaining 80% for training and the remaining 20% for testing. The whole dataset is divided into these pieces in order to reserve an adequate amount of data for training and testing while avoiding issues with over- and under-fitting. The centroids for the various data points are assessed as feature vectors using the training dataset to train the RD-CNN algorithm. The testing dataset is used to test the framework once it has been trained. This evaluates the various assessment measures as shown in Algorithm 1.
**Algorithm 1**: Our Modified Residual-Dense CNN Network Architecture
Step 1:Input: Training dataset, X; Corresponding labels, Y; Number of residual blocks, num_res_blocks Number of dense blocks, num_dense_blocks; Number of layers within each residual block, num_res_layers; Number of layers within each dense block, num_dense_layersStep 2: Output: Trained Residual-Dense Network modelStep 3:[Initialization]: a. Define the input layer: Input layer: $X_{Input}$, shape determined by dataset b. Add an initial convolutional layer: Initial Convolutional Layer: $X_{conv}=Conv1D\left(X_{Input},\;filters=63,\;kernel=3\right)$ c. Add residual-dense blocks: Initialize $X_{current}=X_{conv}$
Step 4: [Define Inner Residual Block]: Repeat For k = 1 to num_res_blocks do: a. Initialize a new Block: Block input: $X_{block\_input}=X_{current}$ b. Repeat For I = 1 to num_res_layers do Convolutional layer: Convolutional Layer:   $X_{Conv\_i}=Conv1D\left(block_{Input},\;filters=63,\;kernel=3\right)$ Batch normalization:  $Bn\_i=BatchNormalization\left(X_{Conv\_i}\right)$ Activation:  $act_{i}=ReLU\left(Bn_{i}\right)$ c. Residual connection: Residual output: $X_{residual}=X_{act\_num\_res\_layers}+X_{Current}$ d. Update $X_{current}=X_{residual}$
Step 5:[Define the Dense Block]: Repeat For k =1 to num_dense_blocks do: a. Initialize a new Block: Block input: $X_{block\_input}=X_{current}$ b. Repeat For I= 1 to num_dense_layers do Convolutional layer: Convolutional Layer: $X_{Conv\_i}=Conv1D\left(block_{Input},\;filters=63,\;kernel=3\right)$ Batch normalization:  $Bn\_i=BatchNormalization\left(X_{Conv\_i}\right)$ Activation:  $act_{i}=ReLU\left(Bn_{i}\right)$ c. Concatenate with block input: output: $X_{concat}=\mathrm{Concatenate}\;\left(X_{block_{input}},X_{num\_dense\_layers}\right)\;$ d. Update $X_{current}=X_{concat}$
Step 6:Global Average Pooling and Fully Connected Layers: Global average pooling: $avg\_pool=GlobalAvgPooling(X_{current}$) Flatten layer: $flt\_layer=Flatten(avg\_pool_{\;})$ Fully-connected layers: $dense=Dense(flt\_layer{,}_{\;}units=64,\;activation=ReLU$)Step 7:[Return Trained Residual-Dense Network model]

### 3.5. Proposed Residual-Dense CNN Model

Algorithm 1 provides a summary of the many phases of our suggested architecture. The provided algorithm outlines the construction and training process of a modified Residual-Dense CNN Network architecture for tasks such as classification or regression. It begins by initializing the model with an input layer and an initial convolutional layer to extract initial features. Residual-dense blocks are then introduced, where each block combines the advantages of residual and dense connections. Within each residual block, convolutional layers are applied, followed by batch normalization and ReLU activation. The final output of a residual block is added to its input, creating a residual connection. Similar operations are performed in dense blocks, where the outputs of convolutional layers are concatenated with the initial input. A global average pooling layer aggregates spatial information, followed by fully connected layers for feature extraction. The model is compiled with a specified loss function, optimizer, and evaluation metrics, then trained on the input data. The trained model is then returned. This algorithm provides a structured and formalized guide for creating and training the proposed network architecture. Our algorithm’s inputs include a collection of ECG signals, labels for those ECG signals, and the number of arrhythmia classifications, while the assessed performance metrics are output by our algorithm.

Incorporating residual and dense connections into the RD-CNN model is a strategic architectural choice that aims to solve some of the most important problems in training deep neural networks, especially in the area of detecting arrhythmias from ECG signals. This integration is made to improve the flow of information, stabilize gradient propagation, and promote the effective reuse of features. All of these things are important for finding arrhythmia patterns in ECG data quickly and accurately.

Residual connections, initially popularized by the ResNet architecture, play a pivotal role in facilitating seamless information flow throughout the network. By allowing the model to learn residual mappings—the difference between the predicted output and the desired outcome—these connections enable a more efficient transfer of information across layers. In the context of ECG signal analysis, where preserving and transmitting relevant information is paramount, residual connections prove invaluable. They effectively mitigate the vanishing gradient problem that often plagues very deep networks, allowing gradients to propagate back through the layers with greater ease. This aspect is particularly crucial for the RD-CNN model’s ability to capture intricate patterns within ECG signals.

Furthermore, the RD-CNN model harnesses the power of dense connections, a hallmark of dense blocks, to foster enhanced feature reuse and cross-layer interaction. The essence of a dense block lies in its ability to endow each layer with direct access to the feature maps generated by all previous layers. In return, each layer contributes its own feature maps to the subsequent layers. This interconnection between layers enables a robust pathway for gradient flow, effectively addressing challenges related to gradient stability. In the RD-CNN architecture, the amalgamation of dense connections with residual connections creates a formidable conduit for gradient propagation. This synergistic effect, in turn, enhances the stability of gradients during the model’s training process. This improved gradient stability is a key part of how the network is able to learn complex patterns from ECG signals, which leads to more accurate and useful solutions.

By combining residual and dense connections, there is also a strong focus on reusing features, which is important for complicated signal analysis, such as figuring out what an ECG means. Dense connections, by design, facilitate the exchange and amalgamation of features learned across various layers. This dynamic feature reuse mechanism enables the RD-CNN model to capture a spectrum of information at different scales and levels of abstraction. In the realm of ECG signal analysis, where the relevance of different signal components varies across scales, this architectural approach proves highly advantageous. The model’s capacity to exploit diverse features at multiple levels equips it to discern both local and global patterns within the ECG signals. So, the RD-CNN model is likely to improve the accuracy of detection by accurately capturing the subtle patterns that are associated with arrhythmia.

As described in Algorithm 1, we first employ the signal resizing procedures. Those signals are then sent through a RD-CNN model. In this Section 3.4, the architecture’s specifics are covered. Algorithm 1 is then used to process the characteristics that were extracted. The different convolutional neural network architecture hyperparameters employed in the suggested model: Hyperparameter Value or Description Convolutional Layers: How Many 6 Max Pooling Layers: Number 6 Convolutional Layer Kernel Size 3 × 3 Pool Size for Maximum Layer Pooling 2 × 2 Steps 2 Activation Mechanism 0.5 ReLU Dropout Rate; the arrhythmia signals into the appropriate groups using the proposed approach. In Algorithm 1, our modified residual-dense CNN Network architecture is described, which is explored next in more detail. Finally, evaluations and returns are made for the different performance measures.

The input of the network as x and the output as y. The network consists of multiple residual-dense blocks, interleaved with optional pooling layers for downsampling. Each residual-dense block incorporates both residual connections and dense connections to facilitate effective feature learning and representation. The combination of residual and dense blocks can be represented as follows:

A residual block takes an input *x* and produces an output *y*. It can be represented mathematically as:

(1) Y=F(x)+X

where *F*(*x*) represents the residual mapping learned by the convolutional layers within the residual block.

A dense block takes an input *x* and produces an output *y*. It can be represented mathematically as:

For each layer l in the dense block:

(2) $$Y_{i}=H_{i}\left(y_{0},y_{1},y_{2},\ldots \right)$$

where $\left(y_{0},y_{1},y_{2},\ldots \right)$ represents the concatenation of the outputs from all previous layers within the dense block, and $H_{i}$ denotes the composite function applied to the concatenated feature maps. The residual-dense block combines the concepts of residual blocks and dense blocks. It integrates both residual and dense connections within each block to promote effective information flow and capture feature hierarchies efficiently.

The mathematical representation of a residual-dense block can be expressed as:

For each layer l in the residual-dense block:

(3) $$Y_{i}=F_{i}\left(y_{0},y_{1},y_{2},\ldots \right)+Y_{i-1}$$

where $y_{0},y_{1},y_{2},\ldots$ represents the concatenation of the outputs from all previous layers within the dense block, $F_{i}$ denotes the residual mapping learned by the convolutional layers within the residual-dense block, and $Y_{i-1}$ represents the output of the previous layer. The residual-dense blocks are stacked together, and the final output *y* is obtained by passing the output of the last residual-dense block through a global average pooling layer and fully connected layers.

Finally, the features are classified by a linear support vector machine (LSVM) model into a five-classes-based heartbeat.

### 3.6. Classification by LSVM

The overall algorithm to recognize the multiclass of heart disease, a dense and residual-based CNN model, along with a linear support vector machine (LSVM), is presented in the form of Algorithm 2. The dense and residual-based CNN model is already explained in the previous section. Here, we explain the LSVM-ML technique.
**Algorithm 2**: Overall algorithm for Classification of five-classes recognition of ECG signals using residual and dense based CNN and LSVM techniques
Step 1:Input: ECG signals dataset, 𝐷; Labels, 𝐿, Number of arrhythmia classes, 𝑛 Step 2:Output: Evaluated performance metrics Step 3:Pre-process ECG signals to remove noise and make classes balance. Step 4:TrainedClassifier = Residual-Dense-Network (n) Step 5:Extract-features by Residual-based dense CNN model ()Step 6:TrainedClassifier by linear support vector machine (LSVM, n)Step 7:ClassifiedLabels = Predicted (TrainedClassifier) Step 8:PerformanceMetrics = EvaluatePerformanceMetrics(ClassifiedLabels, TestingLabels) 
return PerformanceMetrics 

Modern categorization methods include support vector machines (SVM). It has been demonstrated to outperform their competitors in terms of accuracy and computational benefits. It has been used to solve numerous biological categorization issues effectively. The method operates as follows. Consider a collection of points that are individually assigned to one of two classes and are shown in a high-dimensional environment. A hyperplane that maximizes the margin between the two classes of samples is computed using an SVM. The decision boundary is the best hyperplane. To make it multi-class problems, we have used a one-versus-all approach.

Formally, let *n* training samples and their corresponding class labels be denoted by *x*_1_, *x*_2_, …, *x*_n_ and *y*_1_, *y*_2_, …, *y*_n_, respectively. Allow *y*_*i*1_, 1 to stand for the labels of two classes. A linear classifier’s decision boundary may be expressed as *wx* + *b* = 0, where *w* and *b* are model parameters. The margin d can be expressed as *d* = 2/||*w*||^2^ by rescaling the parameters *w* and *b*. The hyperparameters for multiclass SVM are defined as Kernel: RBF, Gamma: 1 × 10^−4^, and C is between 1 to 10.

### 3.7. Hyperparameter Tuning

Hyperparameters are the requirements for a model’s architectural design. The performance of a machine learning or deep learning model depends on the choice of an appropriate set of hyperparameters. Hyperparameter tuning is the process of selecting the best hyperparameters. In this work, the tuning of the hyperparameters is carried out using the Grid Search heuristic. It is a method that repeatedly searches through a set of hyperparameters, testing out different combinations at random to obtain the best result. The hyperparameters that produce the greatest values for accuracy are chosen. The model can train on the best parameters without aliasing thanks to our use of the random search strategy for hyperparameter selection. In order to compare the suggested framework’s performance fairly, we tuned the hyperparameters of all the comparing techniques employed in this study. After tuning hyperparameters, we have adjusted the following parameters as displayed in Table 4.

The optimal value for each hyperparameter is determined by maximizing accuracy while minimizing computation time. Hyperparameters batch size of tested values (16, 32, 64, 128), optimized value (16), dropout Value-tested values (0.2, 0.3, 0.40) optimized value (0.3), learning Rate test values (0.0001, 0.001, 0.01) optimize value (0.001), and Optimizer-tested techniques (SGD, Adam) selected Adam. The Hyperparameter tuning process was systematic through Grid search technique and aimed at finding the best trade-offs between accuracy and computation time. These optimized hyperparameters enhanced the performance of a Residual-Dense CNN model and allow it to better handle ECG data for arrhythmia detection tasks.

## 4. Experimental Analysis

We use our own experimental analysis to evaluate the performance of the proposed architecture compared to state-of-the-art approaches. On every dataset specified in Section 3.1, we conduct simulations, and we assess every evaluation measure mentioned in Section 3.2. We conducted a comparison of many machine learning techniques. This aids in our comprehension of how well our strategy performs in comparison to other standard choices in terms of machine and deep learning algorithms. We also conduct ablation research to examine how our strategy performs when we apply other strategies.

The computer language Python was used exclusively to implement the code. To support the experimental investigation, we employed a number of Python packages, including sklearn, TensorFlow, NumPy, Pandas, etc. Additionally, we used a few GitHub repositories that were open to the public. On a laptop equipped with an Intel i7 11th generation CPU, 32 GB of RAM, and a graphics card, all the simulations were carried out.

### 4.1. Evaluation Metrics

We outline the numerous assessment measures we utilized in this part to judge the effectiveness of the job we had in mind. We have employed a few common assessment indicators for this study. The different assessment measures are briefly described here. 1. *Accuracy (ACC)* indicates whether the classifier correctly assigned the data points to the appropriate classes. Formally, it may be expressed as the proportion of all samples that were properly categorized to all samples. Equation (4) can be used to describe it mathematically.

(4) $$Accuracy\;\left(ACC\right)=\frac{T\;P+T\;N}{T\;P+T\;N+F\;P+F\;N\;}$$

*Sensitivity (SE)* is crucial when the consequences of missing positive cases (false negatives) are significant, such as in medical diagnosis. A high sensitivity indicates that the model is effective at detecting true positives as calculated by Equation (5).

(5) $$Senstivity\;\left(SE\right)=\frac{TP}{TP+FN}$$

*Specificity (SP)* is important when the consequences of misclassifying negative cases (false positives) are significant. High specificity indicates that the model is good at avoiding false positives and it is calculated by Equation (6) as:

(6) $$Specificity\;\left(SP\right)=\frac{TN}{TN+FP}$$

The f1−score is another important metric used to evaluate the performance of classification models, particularly when dealing with imbalanced datasets. It combines precision and recall (sensitivity) into a single value and provides a balance between the two. The F1 score is calculated by Equation (7) as:

(7) $$f1-score=2\times \frac{Precision\times Recall}{Precision+Recall}$$

where *Precision (PR)* and *Recall (RE)* are calculated by Equations (8) and (9) as:

(8) $$Precision\left(PR\right)=\frac{TP}{TP+FP}$$

(9) $$Recall\;\left(RE\right)=Senstivity=\frac{TP}{TP+FN}$$

In the examples above, True Positive (*TP*) stands for heartbeat samples that were classified as heartbeat, False Positive (*FP*) for non-heartbeat samples that were classified as correct heartbeat, True Negative (*TN*) for non-heartbeat samples that were classified as non-heartbeat, and False Negative (*FN*) for heartbeat samples that were classified as non-heartbeat samples.

*Kappa*, also known as Cohen’s Kappa, is a statistical metric that assesses the level of agreement between the predicted classifications of a model and the actual classifications. It takes into account the agreement that might occur by chance, making it a valuable metric, especially for cases where the distribution of classes is imbalanced or when assessing inter-rater reliability. The Kappa coefficient is calculated using the formula:

(10) $$Kappa=\frac{P_{o}-P_{e}}{1-P_{e}}$$

where $P_{o}$ is the observed agreement, i.e., the proportion of cases where the model’s predictions match the actual classes. The parameter $P_{e}$ is the expected agreement, representing the agreement that would be expected purely by chance. The *Kappa* coefficient can range from −1 to 1.

**Figure 5:**
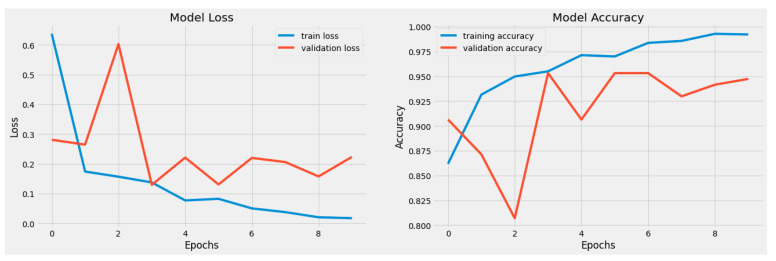
Training and Testing Accuracy Loss based on 6 to 10 epochs.

**Figure 6:**
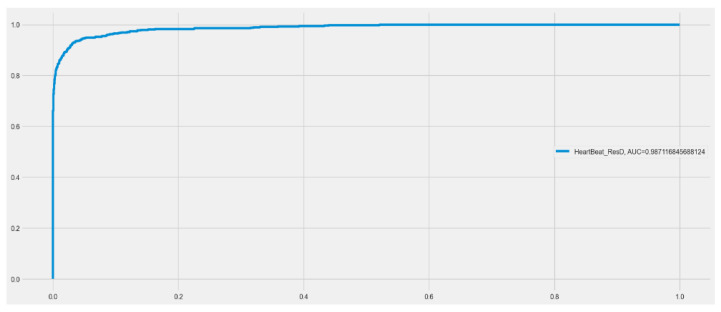
Area under the curve (AUC) for proposed RD-CNN model to recognize cardiac health.

Both the original dataset utilized in the proposed model and our resized dataset were used in the experiments. The cross-entropy loss function and metrics during model training are shown in Figure 5 for the RD-CNN model. In practice, cross-entropy loss for the classification of cardiac health patterns is a common and effective approach. Cross-entropy loss, also known as log loss, is a suitable choice for classification tasks, including those related to diagnosing cardiac health conditions based on patterns in medical data. The performance of the CNN network on the heartbeat dataset is shown graphically in (a) loss values (training and validation) and (b) accuracy graphs (training and validation) with respect to epochs. In addition, the overall AUC curve is also displayed in Figure 6. On average, this AUC curve shows the overall achievement of the proposed RD-CNN architecture.

### 4.2. Comparison with Various Systems

On the MIT-BIH and PTB ECG datasets, we compare the performance of our suggested Residual-Dense-CNN with a number of recent standard state-of-the-art approaches in this section. Table 4 tabulates the findings acquired in terms of the 5-fold cross-validation test. As can be seen from Table 5, our suggested Residual-Dense-CNN approach performs much better than the other state-of-the-art techniques in terms of the values attained for parameters, accuracy, AUC, kappa, and F1 score. We have compared the RD-CNN model with other techniques such as AlexNet-SVM [13], CNN-filtering [16], SVM [21], CNN-LSTM [24], RNN-LSTM [31], DeepCNN [33], and CNN-Pool [34]. Compared to other approaches, as mentioned in Table 1, we have selected these studies because they are easy to implement and because these techniques detect multiclass heart disease.

We have implemented these techniques as described in the corresponding studies. The AlexNet-SVM [13], CNN-filtering [16], SVM [21], CNN-LSTM [24], RNN-LSTM [31], DeepCNN [33], and CNN-Pool [34] techniques are used for comparison to detect and classify in similar training and testing configurations. The different variants of these studies have been implemented according to the methodology designed for the given dataset.

Table 5 provides a comprehensive overview of the comparative performance results, and it is evident that our RD-CNN model demonstrates a clear advantage over the other evaluated techniques. These results back up the idea that combining residual and dense connections in the RD-CNN architecture makes it much easier to spot arrhythmias. This is shown by higher AUC values, more stable Kappa statistics, and better F1 scores.

Overall, the presented results firmly establish our Residual-Dense Convolutional Neural Network as a promising advancement in the field of ECG-based arrhythmia detection. The comparative analysis highlights the RD-CNN’s superiority in terms of multiple evaluation metrics, validating its potential to enhance the accuracy and efficiency of arrhythmia detection in clinical settings and indicating its capacity to outperform existing state-of-the-art techniques.

To compare the models, we used the same implementation as described in the corresponding papers. The deep-learning algorithms outperform traditional classification methods like SVM by a small margin. However, they continue to perform poorly when compared to our suggested RD-CNN approach. We see that the Residual-Dense-CNN strategy once again outperforms the competition in terms of accuracy for the ECG datasets. While it is the best performer for the F1 score. The analysis shown above demonstrates that our suggested Residual-Dense-CNN approach beats a number of standard machine learning algorithms.

**Figure 7:**
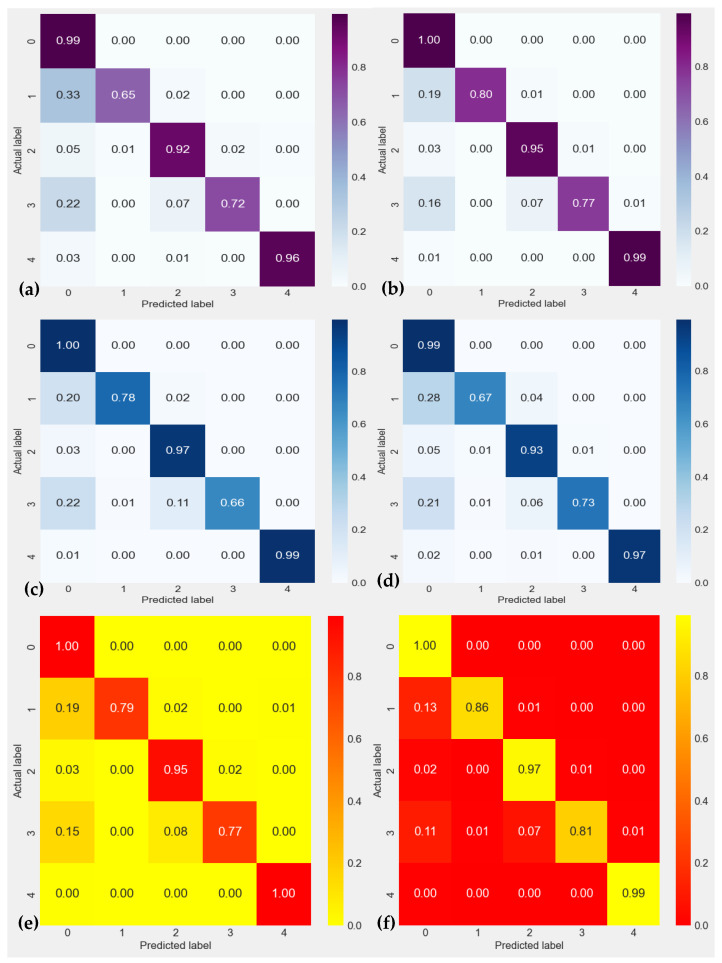
Confusion matrix of ECG heartbeat classification results for testing set with the inter-patient scheme in terms of 0: Normal Beat, 1: Supraventricular Premature Beat, 2: Premature Ventricular Contraction, 3: Fusion of Ventricular and Normal Beat, and 4: Unclassifiable Beat. In this figure, (**a**) shows the CNN-LSTM [24], (**b**) represents the RNN-LSTM [31], (**c**) displays the DeepCNN [33], (**d**) shows the CNN-Pool [34], (**e**) CNN-filtering [16] and (**f**) SVM [21] techniques.

In addition to comparing the performance of our new Residual-Dense-CNN methodology with known modern methods for cardiac heartbeat identification from ECG data, we also compare it with state-of-the-art techniques with our proposed preprocessing of ECG data. Results are mentioned in terms of the confusion matrix as displayed in Figure 7. According to Figure 7, our suggested Residual-Dense-CNN strategy outperforms all other methods for the ECG dataset in terms of accuracy. It performs compared to below: (a) shows the CNN-LSTM [24], (b) represents the RNN-LSTM [31], (c) displays the DeepCNN [33], and (d) shows the CNN-Pool [34] techniques. We find that our suggested method performs better than every other modern method for the ECG Dataset in terms of accuracy and precision. The explanation above shows that the technique we have suggested produces accurate and consistent results across all performance indicators for both datasets.

**Figure 8:**
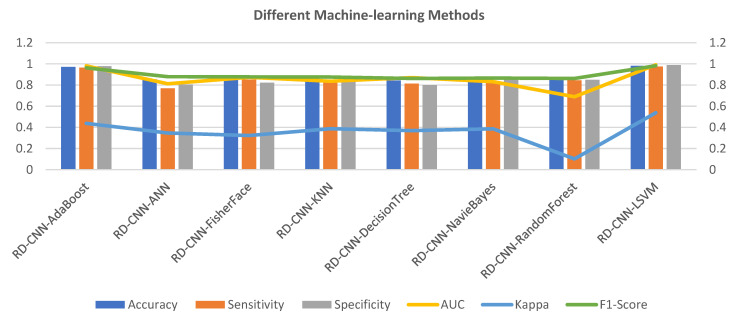
Various machine-learning algorithms comparison with the proposed RD-CNN model to recognize multiclass cardiac health.

Figure 8 provides the evaluation results of various machine learning (ML) algorithms applied to a classification task. Each algorithm’s performance is assessed using multiple metrics, offering insights into its effectiveness for the given task. Among the methods evaluated, “RD-CNN-AdaBoost” emerges as a standout performer in terms of accuracy, achieving an impressive score of 0.971. This indicates that the model made correct predictions for a significant portion of the dataset. Additionally, this algorithm demonstrates a high level of sensitivity (0.965) and specificity (0.978), showcasing its ability to accurately identify both positive and negative instances. The Area Under the ROC Curve (AUC) value of 0.981 further confirms its strong discriminative power, while the F1 Score of 0.962 highlights a good balance between precision and recall. However, it is important to note that the Kappa value of 0.438 suggests only a moderate level of agreement beyond what could be expected by chance.

Comparatively, the RD-CNN-ANN algorithm shows a lower accuracy of 0.855. Although its sensitivity and specificity values are notably lower than those of the top-performing algorithm, it still manages to achieve a relatively high F1 Score of 0.879, indicating that it balances precision and recall reasonably well. Its AUC value of 0.812 suggests decent discriminatory ability. However, the lower Kappa value of 0.347 indicates a weaker level of agreement compared to other methods. The RD-CNN-NavieBayes achieves an accuracy of 0.854 with balanced sensitivity and specificity. Its AUC of 0.832 is reflective of its ability to distinguish between classes. While the F1 Score of 0.866 indicates a fair balance between precision and recall, the Kappa value of 0.386 suggests only moderate agreement.

The RD-CNN-RandomForest attains an accuracy of 0.879, with notable sensitivity and specificity values. However, its AUC of 0.689 is comparatively lower, possibly indicating room for improvement in terms of discrimination. The Kappa value of 0.102 suggests poor agreement, which might raise concerns about its reliability beyond random chance. Lastly, the RD-CNN-LSVM showcases remarkable results across the board. It achieves an accuracy of 0.981 with high sensitivity (0.975) and specificity (0.988) values, implying a robust ability to accurately classify instances. Its AUC value of 0.991 is indicative of outstanding discriminative power. Moreover, the Kappa value of 0.538 signifies a substantial level of agreement, strengthening its reliability.

The RD-CNN-AdaBoost and “RD-CNN-LSVM” algorithms outperform the others in terms of accuracy, sensitivity, specificity, AUC, and Kappa, making them strong candidates for this classification task. The RD-CNN-ANN, RD-CNN-FisherFace, RD-CNN-KNN, and “RD-CNN-DecisionTree exhibit competitive but varied performances, with differences in precision-recall trade-offs and agreement levels. “RD-CNN-NavieBayes” and “RD-CNN-RandomForest” demonstrate moderate results overall, with notable strengths and areas for improvement. Due to the high computational time of the RD-CNN-AdaBoost classifier, we have used a linear support vector machine (LSVM) in this paper to recognize multiple classes of heart diseases.

### 4.3. Ablation Study

In this part, we conduct an ablation research to examine how the various elements of our suggested approach affect the outcomes. In our suggested paradigm, we investigate the impacts of denoising and data imbalance based on five-classes of cardiac heart diseases.

**Figure 9:**
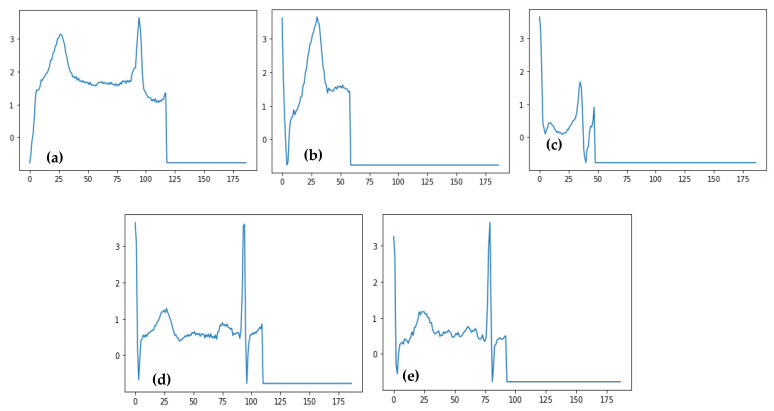
Predicted ECG signals related to four categories of heartbeat such as (**a**) Normal Beat, (**b**) Supraventricular Premature Beat, (**c**) Premature Ventricular Contraction, (**d**) Fusion of Ventricular and Normal Beat, and (**e**) Unclassifiable Beat.

We assessed different outcomes for the following settings: residual-dense-CNN without denoising and class-imbalance; residual-dense-CNN without denoising; residual-dense-CNN without class-imbalance; residual-dense-CNN without denoising; and proposed residual-dense-CNN (with denoising). The results, which were obtained using both datasets, are shown in Figure 9 for both ECG datasets, such as PTB and MIT-BIH. The results show that, when compared to the other approach versions, the suggested Residual-Dense-CNN, which combines denoising and class-imbalance, performs the best. For the ECG dataset, Residual-Dense-CNN without denosing and Residual-Dense-CNN without class-imbalance produce poor results when just one component is removed. This demonstrates the importance of the class-imbalance technique’s complementary impact when used with denoising. The performance of our approach suddenly declines when all two components are taken away, demonstrating the significance of these parts. The description above highlights the relative significance of each element, their interdependence, and their value in attaining the best outcomes for arrhythmia diagnosis from the ECG data.

Table 6 presents a comparison of the results obtained from three different models for recognizing five classes of heart diseases, focusing on their utilization of dense and residual blocks. The models evaluated are the RD-CNN, Dense-CNN, and the newly proposed RD-CNN. The RD-CNN model, with 25.4 million parameters, achieves an accuracy of 86.0%. It demonstrates a sensitivity of 82.0%, specificity of 84.2%, an AUC of 0.816, a Kappa coefficient of 0.287, and an F1 Score of 0.835. The Dense-CNN model, comprising 7.9 million parameters, achieves an accuracy of 84.0%. It displays a sensitivity of 77.3%, specificity of 85.2%, an AUC of 0.860, a Kappa coefficient of 0.379, and an F1 Score of 0.872. The proposed RD-CNN, featuring 6.4 million parameters, stands out with remarkable performance, achieving an accuracy of 98.1%, sensitivity of 97.5%, specificity of 98.8%, an AUC of 0.991, a Kappa coefficient of 0.538, and an F1 Score of 0.982. These results collectively indicate that the proposed RD-CNN model outperforms the other two models in terms of accuracy and other crucial evaluation metrics, making it a promising candidate for accurate heart disease classification.

Compared to other heart diseae patterns (Normal Beat, Supraventricular Premature Beat, Premature Ventricular Contraction, and Unclassifiable Beats), the fusion of Ventricular and Normal Beat are difficult to recognize and so, the classification accuracy is decreased by the proposed method. Therefore, we would like to address this point of -view in future.

## 5. Discussions

The proposed work is based on the training of machine learning (ML) techniques to annotate large and diverse datasets and is still very time-consuming and error-prone. In fact, machine learning techniques whose training does not require extensive and well-annotated datasets are gaining popularity. Consequently, it is feasible to correctly identify and classify cardiac cycle abnormalities (e.g., uncommon cardiologic disturbances) using the limited data available in ECG datasets. Due to the prevalence of cardiac diseases, the classification of heartbeats from digital tracings of ECG signals containing imbalanced datasets is difficult. This study looks at the common problems with classifying images by using a residual-based CNN transfer learning paradigm to divide ECG heartbeats into five stages based on supervised information. This study paper suggests a multichannel RD-CNN design for classifying cardiac beats so that the effectiveness of the RD-CNN architecture can be tested on a smaller dataset. The four-class classification performance of the suggested architecture was satisfactory. The visualization outcomes further demonstrate that the model is capable of differentiating between all classes. As a result, cardiologists can easily classify the heartbeat classes with the use of this model. The following are the key causes of this model’s superior performance:

1. The suggested design makes use of preprocessed photos rather than raw ones. As a result, the suggested architecture lacks high-density regions like the pectoral muscle and tags on mammograms, which are useful for improving classification accuracy.
2. To boost the caliber of training data, the suggested method employs preprocessing steps to enhance the ECG signals.
3. To enable the network to concentrate on a wider range of features information from ECG signals, the model incorporates residual and dense blocks to extract the signals.

The suggested model has the potential to be used in clinical settings as a useful tool to help cardiologists read and comprehend ECG heartbeat data. The classes of ECG heartbeats taken into consideration in this study may be divided into five primary categories, (a) Normal Beat, (b) Supraventricular Premature Beat, (c) Premature Ventricular Contraction, (d) Fusion of Ventricular and Normal Beat, and (e) Unclassifiable Beat. Future work will include rebuilding the techniques used to operate with several classes (for example, more than five different types of heartbeats), developing the work for real-time application, and continually refining and improving it to boost accuracy. Additionally, the same classification procedure may be applied to other dataset types, such as stress and clinical datasets.

Several people die each year from cardiovascular problems, which are among the most serious illnesses. The most common of these cardiac conditions is arrhythmia. This serves as the justification for creating an automated and effective arrhythmia detection method. In this paper, we offer a method for detecting different phases of arrhythmia from ECG data using a deep learning Residual-Dense-CNN architecture and an LSVM-based architecture. We begin by utilizing the IIR Notch filter and FIR filter to denoise the ECG data. This reduces the extra noise that is caused by them for a variety of reasons, such as instrument or recording faults, etc. Various data augmentation techniques use the denoised signal to create a more balanced dataset and a more universal framework. Then, a deep CNN architecture is used to extract features from the enhanced dataset. The deep-learning technique is then applied to the retrieved features in order to eventually categorize the ECG signals into the appropriate arrhythmia types. To get optimal performance, we properly tune the hyperparameters. On several benchmarked datasets, we run extensive simulations and assess a number of common performance indicators. We evaluate how well our method performs in comparison to several baseline machine learning techniques and a few current arrhythmia detection systems. To examine the impact of different elements of our suggested technique’s components on its performance, we also conducted ablation research. By applying more advanced signal processing techniques and identifying more cardiac illnesses, this research may be furthered.

### Current Limitations

The Residual-Dense CNN (RD-CNN) model, as with any deep learning architecture, has its own set of limitations that can affect its performance, particularly in the context of ECG analysis. While the specifics of the RD-CNN model’s limitations depend on its design and implementation, here are some limitations that could apply:

(1) Deep learning models, including RD-CNN, can be prone to overfitting, especially when trained on small datasets or when model complexity is high. This can lead to poor generalizations about new, unseen data.
(2) The model’s performance can be sensitive to hyperparameter choices, such as the number of blocks, layers, filter sizes, learning rate, etc. Improper tuning of these hyperparameters can lead to suboptimal performance.
(3) If the RD-CNN model is trained on a limited or biased dataset, it might not generalize well to different types of arrhythmias or populations that were not well represented in the training data.
(4) The RD-CNN model might struggle with noisy ECG signals, especially when noise levels are high. The model’s performance could degrade in the presence of various types of noise, such as motion artifacts or electrode contact issues.
(5) Training deep learning models like RD-CNN requires significant computational resources and time, which might not be readily available to all researchers or healthcare institutions.
(6) Depending on the architecture, the RD-CNN model might be computationally expensive, making real-time processing or deployment on resource-constrained devices challenging.
(7) ECG signals can vary significantly due to differences in patient demographics, recording conditions, and underlying medical conditions. If these variations are not well represented in the training data, the model’s performance might decrease.

To specifically address the model’s performance in detecting certain types of arrhythmias or under certain conditions, rigorous testing and validation are necessary. The model’s limitations may vary across different arrhythmias. For example, certain rare or complex arrhythmias might not be well captured by the model if they were underrepresented in the training data. Additionally, conditions that lead to significant signal distortions, such as atrial fibrillation with rapid ventricular response, might pose challenges for accurate detection.

## 6. Conclusions and Future Works

The proposed work is based on the training of machine learning (ML) techniques to annotate large and diverse datasets and is still very time-consuming and error-prone. In fact, ML techniques whose training does not require extensive and well-annotated datasets are gaining popularity. Consequently, it is feasible to correctly identify and classify cardiac cycle abnormalities (e.g., uncommon cardiologic disturbances) using the limited data available in ECG datasets. Due to the prevalence of cardiac diseases, the classification of heartbeats from digital tracings of ECG signals containing imbalanced datasets is difficult. This study investigates the prevalent ECG signal classification problems through a residual-based CNN learning paradigm to classify ECG heartbeats into five stages using supervised information. A RD-CNN algorithm is then used to categorize the ECG data for the various cardiac illnesses using the retrieved characteristics. On two benchmarked datasets, we conducted extensive simulations and assessed several performance measures. The effectiveness of our suggested method for detecting heart disease from ECG data was compared with a number of recently presented algorithms. On average, we have achieved an Accuracy of 98.5%, Sensitivity of 97.6%, Specificity of 96.8%, and an Area under the receiver operating curve (AUC) of 0.99. The obtained results demonstrate that it is lightweight and effective, which qualifies it for continuous monitoring applications in clinical settings for automated ECG interpretation to support the cardiologist. In addition, the effectiveness of the proposed framework was substantiated by encouraging experimental results. This study will significantly advance ECG data mining facilitated by machine learning towards intelligent medical decision support. In this paper, we have optimized the model using the cross-entropy loss function. However, there are several other approaches [35,36] that will be tested in the future.

## Figures and Tables

**Table 1:** Recent state-of-the-art ECG heartbeats recognition systems.

Cited	Methodology	Dataset	Accuracy	Limitations
[13]	SVM + Clustering	Private	92%	Disease patterns are identified but no steps were performed for categorizing those patters. Computationally expensive, extensive preprocessing steps are required.
[16]	Convolutional neural network architecture + Preprocessing to remove noise	PhysioNet	F-score of 82%	Disease patterns are identified but no steps were performed for categorizing those patters. Computationally expensive, extensive preprocessing steps are required.
[21]	SVM	MIT-BIH	90.02%	Single dataset was used. Different disease patterns are identified with signal preprocessing steps. Computationally expensive, extensive preprocessing and segmentation steps are required.
[24]	CNN+LSTM	PTIB	98.95%	Five different classes of heartbeat disease patterns are identified. Computationally expensive and applied on a single dataset.
[25]	Preprocessing and quick training are provided by RF neural networks	MIT-BIH	93.5%	Heartbeat disease patterns are identified. Computationally expensive and accuracy is not up-to-the-mark.
[26]	Pre-processing, QRS complex detection, segmentation, feature extraction, and LSTM-based RNNs	MIT-BIH	99.41%	Single dataset was used. Different disease patterns are identified with signal preprocessing steps. Computationally expensive, extensive preprocessing and segmentation steps are required.
[31]	Deep CNN	MIT-BIH- INCART	98.38% accuracy	Five classes of heartbeat, no data imbalance, training is computationally expensive, and there are no preprocessing steps.
[33]	CNN and LSTM	MIT-BIH	98.41% accuracy	Five classes of heartbeat are identified but computationally expensive and it is not generalized solution.
[34]	CNN and Max-Pool	MIT-BIH	99.06% accuracy	CNNs and max-pooling layers usually expect inputs of fixed dimensions. Dealing with variable-length sequences might require additional preprocessing or custom architectures. NNs tend to require a large amount of labeled data to perform well.

**Table 2:** An ECG five-classes Heartbeats collected from two sources.

Category	N ^1^	S ^1^	V ^1^	F ^1^	Q ^1^	Total
Train data	94,573	3415	6799	752	7642	113,181
Test data	38,218	1256	2678	278	2845	45,275
Total	132,791	4671	9477	1030	10,487	158,456

^1^ N: Normal Beat, S: Supraventricular Premature Beat, V: Premature Ventricular Contraction, F: Fusion of Ventricular and Normal Beat, and Q: Unclassifiable Beat.

**Table 3:** Class distributions of MIT-BIH dataset before and after resampling.

Class Name	# Samples Before	# Samples After
N ^1^	132,791	20,000
S ^1^	4671	20,000
V ^1^	9477	20,000
F ^1^	1030	20,000
Q ^1^	10,487	20,000

^1^ N: Normal Beat, S: Supraventricular Premature Beat, V: Premature Ventricular Contraction, F: Fusion of Ventricular and Normal Beat, and Q: Unclassifiable Beat are classes of cardiac disease heartbeats.

**Table 4:** Hyperparameters Settings used by proposed RD-CNN architecture.

Configuration	Value
Network Optimizer	Adam
Maximum number of epochs	10
Network Batch Size	64
Model Learning rate	0.001
Batch normalization	True
Activation function	ReLU
Loss function	Cross-entropy loss
Layer Drop out	5.00 × 10^−1^
Criteria of Early Stopping	Monitor = val__loss, patience = 5

**Table 5:** Comparison with some CNN networks and proposed RD-CNN model based on acquisition datasets.

Model	Params	Accuracy	Sensitivity	Specificity	AUC	Kappa	F1-Score
AlexNet-SVM [13]	144M	0.877	0.777	0.677	0.803	0.331	0.877
CNN-filtering [16]	149M	0.865	0.678	0.805	0.812	0.347	0.879
SVM [21]	24.9M	0.878	0.771	0.821	0.873	0.323	0.877
CNN-LSTM [24]	26.6M	0.875	0.870	0.872	0.836	0.387	0.875
RNN-LSTM [31]	6.9M	0.862	0.760	0.800	0.870	0.369	0.862
DeepCNN [33]	37M	0.874	0.864	0.854	0.832	0.386	0.866
CNN-Pool [34]	33M	0.869	0.834	0.850	0.689	0.102	0.862
Proposed RD-CNN	6.4M	0.981	0.975	0.988	0.991	0.538	0.982

**Table 6:** Comparison with proposed RD-CNN model in terms of dense and residual blocks to recognize five classes of heart diseases.

Model	Params	Accuracy	Sensitivity	Specificity	AUC	Kappa	F1-Score
RD-CNN	25.4 M	0.860	0.820	0.842	0.816	0.287	0.835
Dense-CNN	7.9 M	0.840	0.773	0.852	0.860	0.379	0.872
Proposed RD-CNN	6.4 M	0.981	0.975	0.988	0.991	0.538	0.982

## Data Availability

All datasets used in this study are available online. MIT-BIH Arrhythmia Dataset [22] available at https://www.physionet.org/content/mitdb/1.0.0/ (accessed on 1 January 2023) and PTB Diagnostic ECG Datasets [24] available at https://physionet.org/content/ptbdb/1.0.0/ (accessed on 1 January 2023).
